# Acupoint catgut embedding attenuates fibromyalgia pain through attenuation of TRPV1 signaling pathway in mouse

**DOI:** 10.22038/IJBMS.2023.71431.15534

**Published:** 2024

**Authors:** Feng-Chen Kao, Chia-Ming Yen, Ming-Chia Lin, Hsien-Yin Liao, Hsin-Cheng Hsu, Yi-Wen Lin

**Affiliations:** 1 School of Medicine, College of Medicine, I-Shou University, Kaohsiung, Taiwan; 2 Department of Orthopedics, E-Da Hospital, I-Shou University, Kaohsiung, Taiwan; 3 Department of Orthopedics, E-Da Dachang Hospital, I-Shou University, Kaohsiung, Taiwan; 4 Department of Anesthesiology, Taichung Tzu Chi Hospital, Buddhist Tzu Chi Medical Foundation, Taichung 42743, Taiwan; 5 School of Post-Baccalaureate Chinese Medicine, Tzu Chi University, Hualien 97004, Taiwan; 6 Department of Nuclear Medicine, E-DA Hospital, College of Medicine, I-Shou University, Kaohsiung 82445, Taiwan; 7 College of Chinese Medicine, School of Post-Baccalaureate Chinese Medicine, China Medical University, Taichung 40402, Taiwan; 8 College of Chinese Medicine, Graduate Institute of Acupuncture Science, China Medical University, Taichung 404332, Taiwan; 9 Chinese Medicine Research Center, China Medical University, Taichung 40402, Taiwan

**Keywords:** Acupoint catgut embedding, Chronic pain, Fibromyalgia, Somatosensory cortex, Thalamus, TRPV1

## Abstract

**Objective(s):**

Chronic pain is considered as pain lasting for more than three months and has emerged as a global health problem affecting individuals and society. Chronic extensive pain is the main syndrome upsetting individuals with fibromyalgia (FM), accompanied by anxiety, obesity, sleep disturbances, and depression, Transient receptor potential vanilloid 1 (TRPV1) has been reported to transduce inflammatory and pain signals to the brain.

**Materials and Methods:**

Acupoint catgut embedding (ACE) is a novel acupuncture technique that provides continuous effects and convenience. ACE was performed at the bilateral ST36 acupoint.

**Results:**

We demonstrated similar pain levels among all groups at baseline. After cold stress, chronic mechanical or thermal nociception was induced (D14: mechanical: 1.85 ± 0.13 g; thermal: 4.85 ± 0.26 s) and reversed in ACE-treated mice (D14: mechanical: 3.99 ± 0.16 g; thermal: 7.42 ± 0.45 s) as well as Trpv1^-/- ^group (Day 14, mechanical: 4.25 ± 0.2 g; thermal: 7.91 ± 0.21 s) mice. Inflammatory mediators were augmented in FM individuals and were abridged after ACE management and TRPV1 gene loss. TRPV1 and its linked mediators were increased in the thalamus (THA), somatosensory cortex (SSC), medial prefrontal cortex (mPFC), and anterior cingulate cortex (ACC) in FM mice. The up-regulation of these mediators was diminished in ACE and Trpv1^-/-^ groups.

**Conclusion:**

We suggest that chronic pain can be modulated by ACE or Trpv1^-/-^. ACE-induced analgesia via TRPV1 signaling pathways may be beneficial targets for FM treatment.

## Introduction

Pain is an unpleasant experience arising from the interplay of physiological, pathological, and psychological factors upon tissue injury. Chronic pain is clinically well-defined as symptoms of pain durable over three months. Pain serves as a warning system to alarm an individual to avoid further damage and seek medical treatment (1). Fibromyalgia (FM) is a representative chronic pain that has increasingly affected individuals, the health care system, the economy, and society. Currently, there are no effective treatments for this condition due to a poor understanding of its fundamental mechanisms (2). 

FM is a multifactorial disease categorized by chronic extensive pain, sleep disruption, and hopelessness. The incidence of FM varies between 1–8% of people, according to the analytical conditions (2). Recently, the American College of Rheumatology published an improved indicative system for FM, which uses a widespread pain index (WPI) and symptom severity scale (SS). The WPI analyzes 19 common body regions with pain over 2 weeks. The SS measures exhaustion, awakening, and mental indications. The new criteria for the diagnosis of FM are WPI ≥ 7 and SS ≥ 5, or WPI 3–6 and SS ≥ 9 for above 3 months (3). The US FDA has permitted 3 medicines for FM. Milnacipran (Savella), Duloxetine (Cymbalta), and Pregabalin (Lyrica) act by modulating neurotransmitter levels to modulate pain transmission (4). 

Acupuncture is an Eastern medicine modality, which implicates the insertion of steel needles at acupoints. Acupuncture and electroacupuncture have been reported to attenuate pain, such as inflammatory, neuropathic, and FM (5-10). The beneficial effects of acupuncture in FM extend to its capacity to diminish anxiety, depression, and sleep disturbance (11). Recently, acupoint catgut embedding (ACE) has been developed to prolong the effects of acupuncture, including acupoint sensation, while remaining acceptable in both Western and Eastern medicine. This novel acupuncture technique uses absorbable sutures, allowing the continuous stimulation of a specific acupoint for weeks until the suture is completely absorbed. Evidence has demonstrated that ACE can induce immune responses, such as neutrophil infiltration and enhanced macrophage phagocytosis because the absorbable catgut is recognized as a foreign protein (12). Its long-term therapeutic effect and lower cost have made ACE a generally utilized intervention in managing acute and chronic pain. Recent articles have indicated that ACE combined with electroacupuncture (EA) can significantly attenuate postoperative pain and decrease analgesic use (13). 

Transient Receptor Potential Vanilloid 1 (TRPV1) was first documented as a receptor of capsaicin, the major constituent of chili pepper. TRPV1 is a cation receptor that would be stimulated by chili pepper, capsaicin, acidic pH, mechanical stress, and noxious heat. In peripheral sites, TRPV1 was greatly expressed in A- and C-type sensory neurons to detect inflammation and painful stimuli. TRPV1 was also reported to exist in the CNS for thermoregulation, synaptic transmission, and detection of inflammation. TRPV1 is expressed in some brain sections, including the prefrontal cortex (PFC), hippocampus, amygdala, thalamus, somatosensory cortex (SSC), hypothalamus, and cerebellum in animals (14-17). Ca^2+^ influx through TRPV1 can stimulate kinases, such as protein kinase A (PKA), PKC, and Ca^2+^/calmodulin-dependent kinase II (CaMKII). These kinases activate mitogenactivated protein kinase (MAPK), and pAkt-pmTOR can further increase transcriptional factors with cAMP-response element binding protein (CREB) and nuclear factor kappa-light-chain-enhancer of activated B cells (NFκB). PKA and PKC have been identified as crucial links in the progression of hyperalgesia after tissue damage by sensitizing peripheral sensory afferents to mechanical and thermal stimuli (14-17). 

There is no study that explored the character of TRPV1 in ACE anti-nociception. Hence, we surveyed the actual outcome of ACE on TRPV1 in the brain regions in cold stress-induced FM in mice. We here verified the suggestion that FM is linked with augmented TRPV1 and linked kinases in the mouse brain. Furthermore, we evaluated if ACE can diminish both mechanical and thermal nociception through suppression of TRPV1 signaling. To address our research questions, we used a murine model of FM inducedced by intermittent cold stress (ICS). We propose that targeting the TRPV1 signaling pathway is beneficial for FM treatment.

## Materials and Method

***Experimental animals***

The consumption of mice was certified by the Institute of Animal Care and Use Committee of China Medical University (Permit no. CMUIACUC-2022-424), Taiwan, subsequent to the Principle of Laboratory Animals (National Academy Press). The current research is indicated in agreement with ARRIVE’s advice. Entirely, 40 female C57BL/6 mice 8–12 weeks old, were performed here, containing 30 intact mice (BioLASCO Taiwan Co., Ltd.) and 10 *Trpv1*^−/−^ mice (loss of *Trpv1* gene) were bought (Jackson Lab, Bar Harbor, ME, USA) and matched with C57BL/6 mice over 10 generations. The rodents were hosted in a 12 hr light/dark period. An example number of 10 mice in each group was designed as the total mice wanted for an α of 0.05 and an influence of 80%. The staffs were unknown to group distribution through the examinations and exploration. Mice were subdivided into 4 groups: Intact mice (Group 1: Intact); Cold stress-induced FM group (Group 2: FM); FM treated with ACE group (Group 3: FM + ACE), and FM in *Trpv1*^-/-^ group (Group 4: FM + *Trpv1*^-/-^). 

***FM model and multiplex enzyme-linked immunosorbent assay (ELISA)***

Rodents were accommodated at 24 ± 1°C before FM induction. In the rodent ICS model, the rodents were placed at 4 ± 1°C (starting from 4 pm -10 am). Rodents were previously transported to a normal temperature for a 30-minute’ duration the next morning. The rodents were previously relocated to the cold room for another 30 min. The challenge of conservational temperatures between 4 ± 1°C and 24 ± 1°C every 30 min induced a murine FM model. This progression was repetitive until 4:00 pm. The rodents were next engaged at 4 °C overnight. The processes were repeated for two days. Intact mice were placed in the same place at room temperature. After all experimental treatment of FM at day 14, the mice plasma was collected by retro-orbital sinus puncture and evaluated through Q-view multiplex ELISA tests (Q-view, CA, USA).

***Acupoint catgut embedding treatment***

ACE mice had received ACE treatment at the bilateral ST36 acupoint at days 0 and 7 (18). Similar to human anatomy, the ST36 acupoint is located at 3 mm under the apex of the patella bone intersecting horizontally 1 mm lateral to the anterior border of the tibia bone at the medium of the tibialis anterior muscle. Sterilized syringe needle 0.6 × 25 mm (Terumo Corporation, Tokyo, Japan), acupuncture needle 0.35 × 40 mm (Suzhou Medical Appliance, Suzhou, China), and brown catgut 0.2 × 4 mm (CP Medical Inc, Georgia, USA) were applied to insert catgut. All rodents were positioned in a chamber with isoflurane for anesthesia. Two-sided ST36 were decontaminated with 75% alcohol and the commercial syringe needle was implanted, followed by the catgut inserted at the 5 mm depth (Abstract of methods shown below). 

**Figure:**
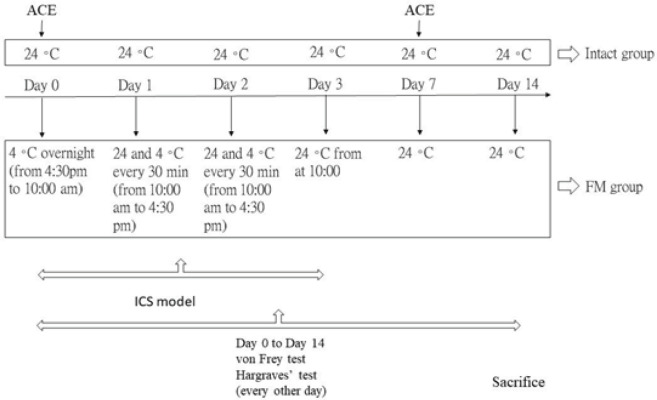
Experimental flow of ICS model

***Pain behavior test***

Animal mechanical or thermal nociceptive manners were measured at 7 intervals from day 0 to 14 (every other day) after FM induction. We first verified the von Frey filament examination, mechanical pain was tested by calculating the strength of replies to stimuli within 3 performances via the electronic von Frey test (IITC Life Science Inc., USA). Rodents were placed in a steel mesh (75 × 25 × 45 cm) and isolated with a plastic cage (10 × 6 × 11 cm). Rodents were confirmed by filament examination at the central zone of the right back paw. The forces were calculated as grams and were documented when the mice withdrew their paw. Besides, the Hargreaves’ test was conducted to measure thermal nociceptive behavior by calculating the time to thermal stimuli with 3 presentations (IITC Life Sciences, SERIES8, Model 390G). The animals were engaged in a cage on top of a glass area. All rodents were placed in the experimental room and provided with environmental enrichment for over 30 min. 

***Western blot analysis***

Rodents were anesthetized with 5% isoflurane and underwent cervical interruption. The thalamus, SSC, ACC, and mPFC samples were separated to remove proteins. Samples were previously engaged and further kept at -80 °C refrigeration. Total extracted proteins were normalized in radioimmunoprecipitation (RIPA) lysis buffer having 50 mM Tris-HCl, 250 mM NaCl, 1% NP-40, 5 mM EDTA, 50 mM NaF, 1 mM Na_3_VO_4_, 0.02% NaN3, and 1× protease inhibitor cocktail (AMRESCO). The total proteins underwent 8% SDS-Tris glycine gel electrophoresis and were transferred to a PVDF membrane. The membrane was blocked with 3% bovine serum albumin (BSA) in TBS-T buffer (10 mM Tris pH 7.5, 100 mM NaCl, 0.1% Tween 20), incubated with a primary antibody in TBS-T for 1 hr at room temperature antibody against anti-tubulin (∼55 kDa, 1:5000, Merck, USA), TRPV1 (∼95 kDa, 1:1000, Alomone, Israel), pPKA (∼40 kDa, 1: 1000, Alomone, Israel), pPKC (∼100 kDa, 1: 1000, Millipore, USA), pPI3K (∼125 kDa, 1:1000, Millipore, USA), pERK1/2 (∼42–44 kDa, 1:1000, Millipore, USA), pp38 (∼ 41 kDa, 1:1000, Millipore, USA), pJNK (∼42 kDa, 1:1000, Millipore, USA), pAkt (∼ 60 kDa, 1:1000, Millipore, USA), pmTOR (∼180 kDa, 1:500, Millipore, USA), pNFκB (∼ 65 kDa, 1:1000, Millipore, USA), and pCREB (∼55 kDa, 1:1000, Millipore, USA), in TBS-T with 1% bovine serum albumin. Peroxidase-conjugated anti-rabbit antibody, anti-mouse antibody, or anti-goat antibody (1: 5000) was utilized as the secondary antibody. The western blot bands were imagined by an improved chemiluminescent substrate kit (PIERCE) with LAS-3000 Fujifilm (Fuji Photo Film Co., Ltd.). The bands’ concentrations of specific molecular weight were quantified with NIH Image J software (Bethesda, MD, USA). α-tubulin was set as the internal controller. The considered percentages were counted by dividing from the same qualified group associated with the intact.

***Immunofluorescence***

Animals were anesthetized and intracardially filled with 0.9 % saline and then 4% paraformaldehyde. The brain was instantly cut apart and further fixed with 4% paraformaldehyde at 4 ºC refrigeration for 3 days. The brains were next engaged in 30% sucrose for protection at 4 ºC for 3 days. The brain was fixed with optimal cutting temperature (OCT) compound and quickly frozen at -80 ºC refrigeration. Frozen brains were sliced at 20-μm thinness and next located on slides. The sections were further nurtured with blocking in 3% BSA, 0.1% Triton X-100, and 0.02% sodium azide, for 1 hr. The brains were then nurtured with primary antibody (1:200, Alomone), TRPV1, and pERK cultured with 1% bovine serum albumin solution at 4 °C overnight. The slices were next nurtured with the secondary antibody (1:500), 488-conjugated AffiniPure donkey anti-rabbit IgG (H + L), 594-conjugated AffiniPure donkey anti-goat IgG (H + L), and Peroxidase-conjugated AffiniPure donkey anti-mouse IgG (H + L) for 2 hr at room temperature. The examples were detected by microscope (Olympus, BX-51, Japan). The pictures were evaluated by Image J software (Bethesda, MD, USA).

***Statistical analysis***

Statistical differences were determined by using the SPSS software. All results are offered as the mean ± standard error (SEM). Shapiro-Wilk test was performed to examine the normality of results. Statistical differences among all clutches were confirmed via the ANOVA test, followed by a *post hoc* Tukey test. Values of *P*<0.05 were considered statistical differences.

## Results

***ACE attenuated mechanical and thermal chronic pain in FM mice***

**Figure 1:**
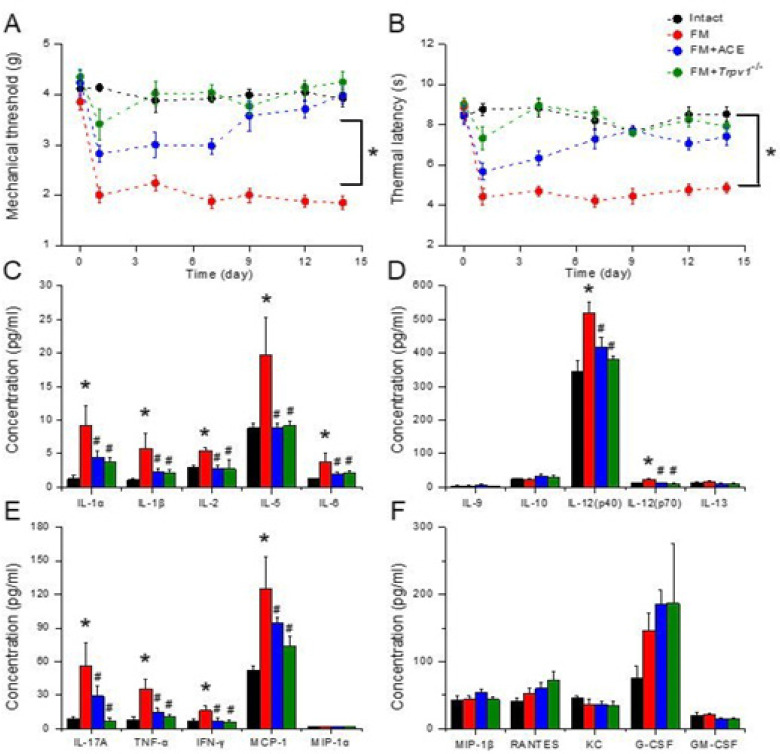
Mechanical, thermal nociception, and inflammatory cytokines in intact, FM, FM+ACE, and *Trpv1*^-/-^ groups

We initially surveyed the possessions of ACE in FM rodents. All rodents had comparable mechanical reactions with normal distribution at baseline before FM induction, without statistical differences among all groups. ICS successfully induced mechanical hyperalgesia, measured using the von Frey test (Figure 1A, red circle, D14: 1.85 ± 0.13, *P˂0.05, n = 10). Mechanical nociception was noticeably relieved by ACE operation and TRPV1 gene deletion (Figure 1A, blue and green circle, D14: 3.99 ± 0.16 and 4.25 ± 0.2, ^#^*P*˂0.05, n = 10, respectively). Furthermore, data from the Hargreaves test indicated major thermal nociception after FM induction (Figure 1B, red circle, D14: 4.85 ± 0.26, *P˂0.05, n = 10). Hyperalgesic latency was attenuated by ACE or *Trpv1* gene loss (Figure 1B, blue and green circle, D14: 7.42 ± 0.45 and 7.91 ± 0.21, ^#^*P*˂0.05, n = 10, respectively).

***Inflammatory cytokines are profoundly augmented in the FM mouse model and can be relieved by ACE treatment or TRPV1 gene deletion***

Because neuroinflammation has been observed in clinical FM patients, we determined the concentration of inflammatory cytokines in mice’s plasma. We performed a multiplex ELISA performance to count the levels of interleukin-1 alpha (IL-1α), IL-1β, IL-2, IL-5, IL-6, IL-9, IL-10, IL-12(p40), IL-12(p70), IL-13, IL-17A, tumor necrosis factor-alpha (TNF-α), interferon-gamma (IFN-γ), monocyte chemoattractant protein 1 (MCP-1), macrophage inflammatory protein 1 alpha (MIP-1α), MIP-1β, regulated on activation, normal T cell expressed and secreted (RANTES), keratinocyte chemoattractant, granulocyte colony-stimulating factor (G-CSF), and granulocyte-macrophage-colony-stimulating factor (GM-CSF). In intact mice, inflammatory cytokines levels were low at baseline. After 14 days of FM induction, IL-1α, IL-1β, IL-2, IL-5, IL-6, IL-12, IL-17A, TNF-α, IFN-γ, and MCP-1 levels were increased (Figures 1C-F, red column, *P<0.05, n = 10). Furthermore, after successions of ACE management, the inflammatory cytokines were markedly attenuated (Figures 1C-F, blue column, *P<0.05, n = 10). Moreover, inflammatory factors were further down-regulated in *Trpv1*^-/-^ mice with ICS (Figures 1C-F, green column, *P<0.05, n = 10).

***ACE or TRPV1 gene deletion attenuated TRPV1 signaling pathways in FM mouse thalamus***

**Figure 2:**
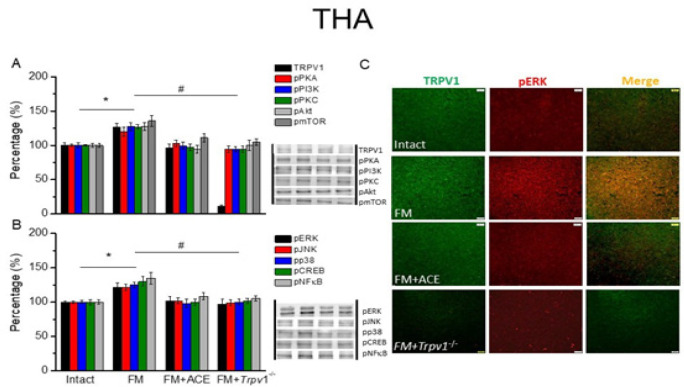
TRPV1 and associated kinases in THA

We then utilized Western blot to quantify proteins in the TRPV1-related mechanism in the thalamus, which receives signals from the spinothalamic tract. TRPV1 was detected in the thalamus of intact mice and was augmented in FM mice (Figure 2A, black column, *P˂0.05, n = 6). ACE consistently diminished overexpression of TRPV1 (Figure 2A, black column, #*P*˂0.05, n = 6). We confirmed an absence of TRPV1 in *Trpv1*^-/-^ mice. 

We next determined the expression of TRPV1-associated protein kinases. Similar to TRPV1, we found that pPKA, pPI3K, and pPKC were augmented in FM mice (Figure 2A, *P˂0.05, n = 6), which were diminished following ACE treatment (Figure 2A, ^#^*P*˂0.05, n = 6) or *Trpv1* gene deletion (Figure 2A, ^#^*P*˂0.05, n = 6). We showed pAkt and pmTOR were augmented in the FM thalamus (Figure 2A, *P˂0.05, n = 6) and decreased in ACE-treated mice (Figure 2A, *P˂0.05, n = 6) and *Trpv1*^-/- ^mice (Figure 2A, #*P*˂0.05, n = 6). Lastly, we found increased pERK, pp38, and pJNK as well as the transcription factors pCREB and pNFκB in the thalamus in FM. These effects were ameliorated by ACE manipulation or *Trpv1* dene deletion (Figure 2B, #*P*˂0.05, n = 6). 

We next used immunostaining for TRPV1 localization in the thalamus. We detected normal TRPV1 in intact mice and augmented in the FM thalamus (Figure 2C, green color, n = 4). ACE consistently relieved overexpression in the FM model (Figure 2C, green color, n = 4). A comparable style was perceived in the *Trpv1*^-/-^ mice (Figure 2C, green color, n = 4). A similar trend was also achieved for pERK (Figure 2C, red color, n = 4). We found TRPV1 and pERK colocalization in wild-type mouse thalamus, a finding that was increased in the FM group. This colocalization was abolished by ACE and *Trpv1*^-/-^ mice (Figure 2C, yellow color, n = 4). Altogether, these discoveries indicate TRPV1 pathways in the FM model, which can be reversed by ACE management and *Trpv1* loss.

***ACE or loss of TRPV1 reduced TRPV1 signaling pathways in FM mouse SSC, ACC, and mPFC***

**Figure 3:**
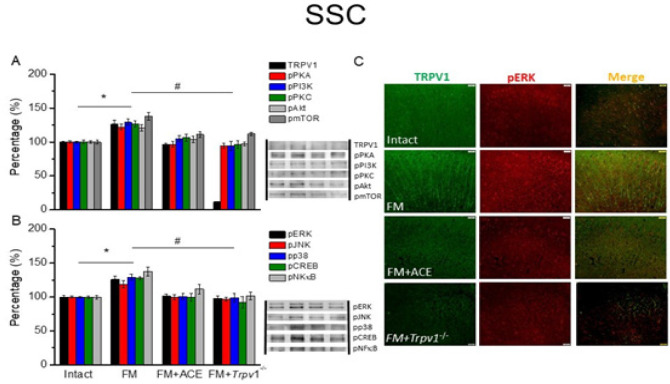
TRPV1 and associated mediators in SSC

**Figure 4:**
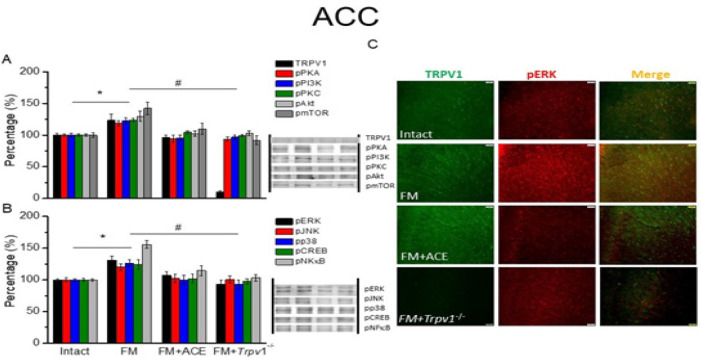
TRPV1 and associated kinases in ACC

**Figure 5:**
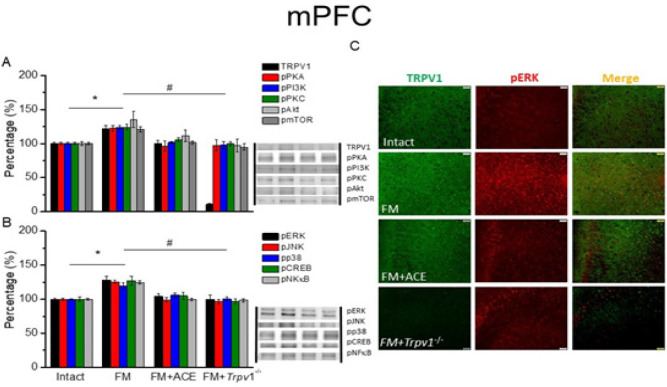
TRPV1 and associated molecules in mPFC

We next quantified TRPV1-associated proteins in cortical areas that govern higher-order pain processing (Figures 3, 4, and 5). Compared to our findings in the thalamus, we found similar patterns of expression of a majority of TRPV1-associated molecules in the SSC, anterior cingulate cortex (ACC), and medial PFC. In particular, our information revealed a statistically significant increase in TRPV1 in the SSC region (Figure 3A, *P˂0.05, n = 6). At 14 days after ICS induction, a remarkable increase in TRPV1 level was observed in the ACC (Figure 4A, black column, *P˂0.05, n = 6). TRPV1 up-regulation was also seen in mPFC (Figure 5A, black column, *P˂0.05, n = 6). These effects were reversed by ACE treatment and *Trpv1* gene loss in the SSC (Figure 3A, ^#^*P*˂0.05, n = 6), ACC (Figure 4A, black column, ^#^*P*˂0.05, n = 6), and mPFC (Figure 5A, black column, 100.4 ± 4.03 %, 10.89 ± 0.82 %, ^#^*P*˂0.05, n = 6). 

Mice with FM had amplified pPKA, pPI3K, and pPKC protein levels in the SSC (Figure 3A, *P˂0.05, n = 6), ACC (Figure 4A, *P˂0.05, n = 6) and mPFC (Figure 5A, *P˂0.05, n = 6). pAkt and pmTOR were increased in rodent SSC (Figure 3A, *P˂0.05, n = 6), ACC (Figure 4A, *P˂0.05, n = 6), and mPFC (Figure 5A, *P˂0.05, n = 6). ACE and *Trpv1* gene loss overturned these effects.

We next determined the expression of MAPK family molecules (pERK, pp38, and pJNK) and transcription factors (pCREB and pNFκB). We observed that these molecules were all increased after FM induction in mice SSC (Figure 3B, *P˂0.05, n = 6), ACC (Figure 4B, *P˂0.05, n = 6), and mPFC (Figure 5B, *P˂0.05, n = 6). These augmented levels were all alleviated after ACE treatment or *Trpv1* gene deletion in SSC, ACC, and mPFC (Figures 3-5B, ^#^*P*˂0.05, n = 6).

Lastly, we used immunofluorescence to measure TRPV1 and pERK intensities in the cortex. Following FM induction, TRPV1, and pERK were amplified in the SSC (Figure 3C, green and red fluorescence, n = 4), ACC (Figure 4C, green and red fluorescence, n = 4), and mPFC (Figure 5C, green and red fluorescence, n = 4). These effects were consistently diminished upon ACE treatment and *Trpv1* gene deletion. Furthermore, colocalization of overexpressed TRPV1 and pERK were detected in the FM mouse SSC (Figure 3C, yellow color, n = 4), ACC (Figure 3C, yellow fluorescence, n = 4), and mPFC (Figure 5C, yellow fluorescence, n = 4). ACE-treated and *Trpv1*^-/- ^mice showed a decrease in TRPV1 and pERK colocalization.

## Discussion

FM is a chronic pain accompanied by complicated symptoms such as anxiety, exhaustion, sleep disruption, and depression. FM is not easy to treat and resistant to opiate or nonsteroidal anti-inflammatory (NSAID) medicines. Recently, antidepressants (Serotonin–norepinephrine reuptake inhibitors: SNRI) and γ-aminobutyric acid (GABAergic) medicines (pregabalin) are first-line used medicines with numerous side effects (19). Vas *et al*. described that acupuncture is of beneficial usage for FM (20). A recent article indicated that acupuncture combined with medicines reduces pain sensations (21). Inflammatory factors both in plasma and cerebral spinal fluid were increased in FM patients (22). Alternative therapies have also been recommended to treat chronic pain such as massage, yoga, exercise, and ACE through various mechanisms (23-25). Researchers reported that ACE and experimental examinations determined the security of ACE. Long-term ACE over 16 weeks significantly diminished body weight suggesting the beneficial treatment of obesity (26). Our recent finding indicated that ACE treatment significantly reduced body weight gain in mice through TRPV1 and its associated molecules (27). ACE also had been suggested to have an obvious effect on climacteric syndrome, in postmenopausal women (28). 

ACE is broadly utilized in the hospital for weight control, insomnia, pain treatment, and osteoarthritis. Recent evidence showed that ACE was highly effective for body weight control (29-31). In a randomized controlled trial, Chen *et al.* embedded sutures in belly acupoints to manage obesity. They found that ACE significantly led to decreased anthropometric measures and enhanced quality of life in obese patients, likely through improvements in the metabolic profile and inflammatory response (30). Duan *et al.* finished a trial to explore the effectiveness of ACE on gallbladder emptying and clinical indications of gallstones. Their data suggest that ACE therapy is effective for gallbladder emptying with a littler treatment course (32). A study determined that ACE has a therapeutic effect on postmenopausal osteoporosis (PMOP) by increasing bone mineral density to alleviate symptoms in PMOP patients (33). In this study, we found that chronic hyperalgesia was induced after cold stress. ACE-treated mice as well as *Trpv1*^-/-^ mice significantly attenuated mice chronic pain. Inflammatory cytokines were also augmented in the FM rodents and were abridged by ACE treatment and *Trpv1* loss. We indicated that TRPV1 pathways were augmented in whole THA, SSC, mPFC, and ACC in FM mice. The increase of these molecules was then diminished in ACE and *Trpv1*^-/-^ groups indicating that chronic pain can be modulated by ACE or Trpv1-/- through TRPV1 path-way.

The use of ACE in musculoskeletal pain has been explored to replace agents such as NSAIDs and acetaminophen, which have severe side effects, including weight gain, gastrointestinal symptoms, and dizziness. A review indicated strong evidence of the effectiveness and safety of ACE for musculoskeletal pain (34). ACE has been conveyed to dramatic intensification of peripheral β-endorphin and reduction of plasma P substance (35). Research proposed that ACE can reliably reduce Complete Freund’s adjuvant-induced inflamed pain by modulating the serotonin 1A (5-HT1A) receptor (36). Moreover, ACE can alleviate the overexpression of N-methyl-D-aspartate receptor (NMDA), pCaMKII, pERK, and pCREB in CFA-treated rats. Intrathecal injection of 5-HT1A receptor agonist mimicked the effects of ACE and can be further blocked by 5-HT1A receptor competitor WAY-100635 (35). Du *et al*. reported that ACE has analgesic effects in withdrawal levels and decreased paw edema in mice inflammatory pain model. ACE markedly attenuated the increase in sigma receptor levels in the mice lumbar spinal cord. ACE also attenuated the increased level of pERK and pp38 and found that injection of sigma receptor agonist PRE084 significantly reversed the phenomena (35). A recent article mentioned that the thalamus is a crucial region of pain signaling in the ascending pathway and is crucial in conveying such syndromes (36). In addition, hyperfunction of the thalamus had been reported in chronic FM pain symptoms. The mPFC is a main brain region that responds to cognitive features of pain processes. Pursuing mPFC is crucial for chronic pain management at a cortical level. Our results indicated that TRPV1 pathways were altered in this region in chronic FM mice, which may regulate complex mental, sensitive, and social behaviors. TRPV1 was indicated to be present in mPFC and triggered by harmful stimulations. We recently determined that downstream of TRPV1 molecules were increased after inflammatory pain in SSC and ACC and can be reversed by EA treatment (5). In the current study, our data showed an increased TRPV1 pathway in the THA, SSC, mPFC, and ACC of mice. These results might provide novel evidence for a potential target of FM therapeutic intervention.

Recently, we demonstrated that EA can relieve both acute and chronic pain through TRPV1 signaling (15-17). Consistent with our hypothesis, cold stress meaningfully initiated mechanical and thermal hyperalgesia, which was reliably reversed by ACE. In addition, chronic pain induction increased the TRPV1 signaling pathway and related molecules in the thalamus, SSC, ACC, and mPFC. Furthermore, ACE dramatically reduced these phenomena, suggesting that TRPV1-induced central sensitization can be reversed. 

(A) Mechanical nociception was obtained from the von Frey filament tests. (B) Thermal nociception observed from the Hargreaves test. (C) IL-1α, IL-1β, IL-2, IL-5, and IL-6. (D) IL-9, IL-10, IL-12(p40), IL-12(p70), and IL-13. (E) IL-17A, TNF-α, IFN-, MCP-1, and MIP-1α. (F) MIP-1β, RANTES, KC, G-CSF, and GM-CSF. *shows statistical consequence when matched to the intact mice. #designates statistical implication matched to the FM

FM: Fibromyalgia; ACE: Acupoint catgut embedding

The signals have four lanes of Intact, FM, FM+ACE, and Trpv1-/- groups. (A) TRPV1, pPKA, pPI3K, pPKC, pAkt, and pmTOR. (B) pERK, pp38, pJNK, pCREB, and pNFκB. *specifies statistical consequence when matched with the intact group. #designates statistical importance when equal to the FM group. n = 6 in all groups. (C) Immunofluorescence labeling of TRPV1, pERK, and dual staining in THA (green, red, or yellow). The scale bar means 100 μm. n = 4 in all groups.

FM: Fibromyalgia, ACE: Acupoint catgut embedding

The blot signals have 4 tracks of protein appearance of Intact, FM, FM+ACE, and Trpv1-/- groups. (A) TRPV1, pPKA, pPI3K, pPKC, pAkt, and pmTOR. (B) pERK, pp38, pJNK, pCREB, and pNFκB protein levels. *specifies statistical consequence compared to the intact rodents. #designates statistical consequence compared to FM rodents. n = 6 per group. (C) Immunofluorescence mark of TRPV1, pERK, and dual-labeling in SSC (green, red, or yellow). Scale bar = 100 μm. n = 4 in all groups.

The blot bands of protein expression in order of Intact, FM, FM+ACE, and Trpv1-/- groups. (A) TRPV1, pPKA, pPI3K, pPKC, pAkt, and pmTOR. (B) pERK, pp38, pJNK, pCREB, and pNFκB protein levels. *shows statistical implication compared to the intact group. #shows statistical consequence compared to FM. n = 6 per group. (C) Immunofluorescence labeling of TRPV1, pERK, and dual staining in the mice ACC (green, red, or yellow). Scale = 100 μm. n = 4 in all groups

The blot bands of protein expression in order of Intact, FM, FM+ACE, and Trpv1-/- groups. (A) TRPV1, pPKA, pPI3K, pPKC, pAkt, and pmTOR. (B) pERK, pp38, pJNK, pCREB, and pNFκB. *indicates statistical implication compared to the intact group. #indicates statistical consequence compared to the FM group. n = 6 per group. (C) Immunofluorescence mark of TRPV1, pERK, and paired staining in mPFC (green, red, or yellow). Scale bar = 100 μm. n = 4 in all groups

**Figure 6:**
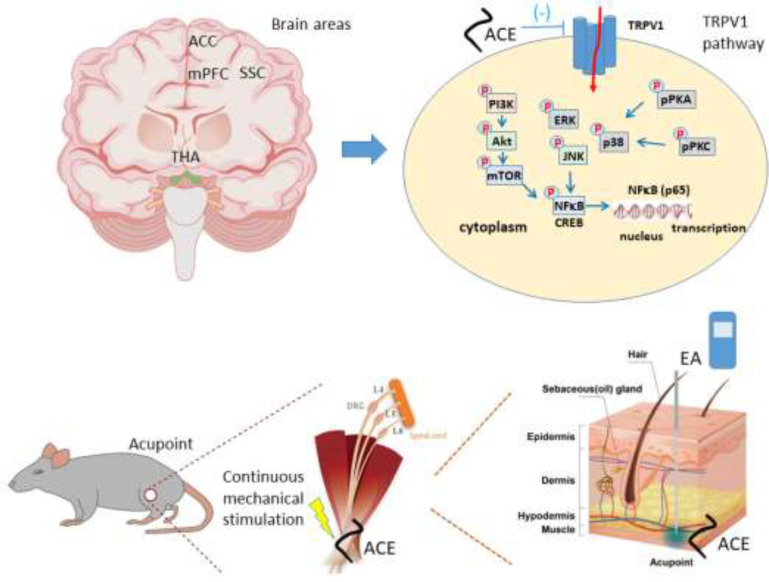
Schematic diagram of TRPV1 and related molecules underlying ACE

The illustrative photograph displays the prominence of analgesic mechanisms of TRPV1 and related factors in FM pain. ACE or Trpv1-/-can significantly attenuate the overexpression of the TRPV1 signaling pathway in the mouse brain.

## Conclusion

We showed that ACE can reliably attenuate thermal stress-induced mechanical and thermal nociception through down-regulation of TRPV1 and linked kinases in a number of sections of the mouse brain (Figure 6). Furthermore, our data suggest the crucial role of TRPV1 signaling in FM pain development, which can serve as a novel target for medicine development.

## Authors’ Contributions

FC K and CM Y performed the experiments and analyzed and interpreted the data. HC H and HY L wrote the draft manuscript. MC L and YW L revised and submitted the manuscript. All authors have read and approved the final manuscript.

## Data Availability

The data used to support the findings of this study are available from the corresponding author upon request.

## Conflicts of Interest

The authors declare no conflicts of interest.
